# One-Step Dry Coating of Hybrid ZnO–WO_3_ Nanosheet Photoanodes for Photoelectrochemical Water Splitting with Composition-Dependent Performance

**DOI:** 10.3390/mi14122189

**Published:** 2023-11-30

**Authors:** Muhammad Shehroze Malik, Deepto Roy, Doo-Man Chun, A. G. Abd-Elrahim

**Affiliations:** 1 School of Mechanical Engineering, University of Ulsan, Ulsan 44610, Republic of Korea; shehrozemalik80@gmail.com (M.S.M.); deepto43.me@gmail.com (D.R.); ahmed_galal@aun.edu.eg (A.G.A.-E.); 2 Physics Department, Faculty of Science, Assiut University, Assiut 71516, Egypt

**Keywords:** ZnO–WO_3_ hybrid photoanodes, charge transfer kinetics, photoelectrochemical water splitting, nanoparticle deposition system (NPDS), nanosheets, nanocomposites, bandgap optimization

## Abstract

In this study, the potential of zinc oxide (ZnO), tungsten oxide (WO_3_), and their composites (ZnO–WO_3_) as photoanodes for photoelectrochemical (PEC) water splitting was investigated. ZnO–WO_3_ nanocomposites (NCs) were deposited on fluorine-doped tin oxide substrates at room temperature using a one-step dry coating process, the nanoparticle deposition system, with no post-processes. Different compositions of ZnO–WO_3_ NCs were optimized to enhance the kinetics of the PEC water-splitting reaction. Surface morphology analysis revealed the transformation of microsized particle nanosheets (NS) powder into nanosized particle nanosheets (NS) across all photoanodes. The optical characteristics of ZnO–WO_3_ photoanodes were scrutinized using diffuse reflectance and photoluminescence emission spectroscopy. Of all the hybrid photoanodes tested, the photoanode containing 10 wt.% WO_3_ exhibited the lowest bandgap of 3.20 eV and the lowest emission intensity, indicating an enhanced separation of photogenerated carriers and solar energy capture. The photoelectrochemical results showed a 10% increase in the photocurrent with increasing WO_3_ content in ZnO–WO_3_ NCs, which is attributed to improved charge transfer kinetics and carrier segregation. The maximum photocurrent for a NC, i.e., 10 wt.% WO_3_, was recorded at 0.133 mA·cm^−2^ at 1.23V vs. a reversible hydrogen electrode (RHE). The observed improvement in photocurrent was nearly 22 times higher than pure WO_3_ nanosheets and 7.3 times more than that of pure ZnO nanosheets, indicating the composition-dependence of PEC performance, where the synergy requirement strongly relies on utilizing the optimal ZnO–WO_3_ ratio in the hybrid NCs.

## 1. Introduction

Photoelectrochemical (PEC) water splitting is a promising technique that involves the direct conversion of sunlight and water into electricity and hydrogen. This method is notably regarded as a simple, economical, and direct energy conversion route toward a sustainable, clean, and green society [1,2,3]. Presently, a major source of energy (80%) on earth comes from fossil fuels, such as oil, natural gas, and coal, which are rapidly depleting and unsustainable, resulting in a drastic change in climate through global warming and other forms of pollution. Therefore, it has become necessary to shift toward green, renewable, and environmentally friendly energy sources such as solar, wind, and geothermal energy to meet global energy demand while minimizing environmental impact and cost [4,5].

Significant efforts have been made to advance metal oxide photoanodes (ZnO, TiO_2_, WO_3_, Fe_2_O_3_, and BiVO_4_) [3,6,7,8,9,10,11], particularly in heterostructured electrode forms, to improve system performance. Meeting the specifications of an efficient PEC water-splitting system using a single semiconductor presents challenges [12,13]. Essentially, the overall performance is based on four key steps: (a) absorption of light and generation of charge carriers; (b) separation of charges; (c) efficient transportation of charges; and (d) extraction of the charge carriers [14]. Therefore, this study focuses on developing heterojunction-based nanocomposites (NCs) for photoanodes, aiming to enhance practical applications in PEC systems. We integrated two semiconductors, ZnO (bandgap = 3.35 eV) [15] and WO_3_ (bandgap = 2.8 eV) [16], using a nanoparticle deposition system (NPDS) [15,17]. ZnO, widely used in photovoltaics applications, offers various advantages like cost-effectiveness, favorable bandgap, high electron mobility (bulk mobility: 200 cm^2^·V^−1^·s^−1^), long life time of minority carriers, and good separation of photogenerated carriers [18]. However, its limited light absorption in the visible-infrared spectrum due to its wide bandgap (~3.35 eV) [19] necessitates a solution. To address ZnO’s light absorption limitation, we combined it with a narrow-bandgap semiconductor, tungsten trioxide (WO_3_). This strategic incorporation aims to expand ZnO’s light absorption range. Tungsten trioxide possesses exceptional properties such as high electron mobility, photostability, and a long hole-diffusion length (~150 nm) [20], making it a good candidate to broaden ZnO’s visible absorbance range. This combination forms ZnO/WO_3_ heterostructures, promising to extend the spectrum of light absorption and enhance the performance of photoelectrochemical applications.

So far, vast efforts have been concentrated on the preparation of hybrid heterostructured photoanodes to achieve a lower bandgap, efficient segregation of photogenerated electron-hole pairs due to appropriate band alignment, and enhanced photoresponse current. D. Barreca et al. [6] prepared ZnO–WO_3_ nano-heterostructures using a multistep vapor phase process. First, ZnO samples were prepared within a reservoir using a 3 h thermal treatment at 550 °C. Second, ZnO was mixed with the WO_3_ layers under optimized conditions: pressure = 0.3 M bar and RF power = 20 W for 3 h. The obtained ZnO–WO_3_ composites achieved a 0.055 mA·cm^−2^ photocurrent at 0.8 V vs. Ag/AgCl in 0.5 M Na_2_SO_4_. Y. Xu et al. [21] prepared ZnO thin films by hydrothermal synthesis at 95 °C for 4 h. WO_3_ was then deposited on the prepared ZnO samples using magnetron sputtering at 1 Pa ambient air pressure and 65 W RF sputtering power. The ZnO–WO_3_ composites obtained in their research achieved a photocurrent of 0.353 mA/cm^2^ at 0.3 V vs. Ag/AgCl in a 0.5 M Na_2_SO_4_ solution. J. Ji et al. [22] prepared WO_3_–BiVO_4_–ZnO using different steps. Tungstic acid and a polyvinyl alcohol solution were dissolved to produce a 0.12 M WO_3_ precursor solution. Then they annealed the solution for 1 h at 500 °C. The same procedure was repeated for BiVO_4_ on the WO_3_ film to obtain the WO_3_/BiVO_4_ films. Finally, a ZnO solution was prepared and deposited onto the WO_3_/BiVO_4_ films using spin coating. In this study, the obtained WO_3_–BiVO_4_–ZnO composite at 1.23 V vs. the RHE exhibited a photocurrent of 0.190 mA/cm^2^ in a 0.5 M Na_2_SO_4_ electrolyte.

The studies described above focusing on the combination of WO_3_ and ZnO to produce heterostructure photoanodes show good performance in neutral electrolytes toward PEC water splitting. However, these synthesis techniques can only be used for small substrates and are limited to research and development, not commercial use. This is the case for several reasons: (a) the considerable time required for fabricating small-sized electrodes through multiple steps to achieve the required nanosized powder, followed by its deposition onto substrates; (b) the use of dangerous reactant chemicals that produce useless toxic secondary products as waste; and (c) the difficulty in scaling up for commercial applications because of the time-consuming above techniques. In comparison, the NPDS has distinct features such as (a) one-step dry coating using the vacuum kinetic spray process at room temperature; (b) no hazardous chemicals or waste; (c) facile production of sizable electrodes for mass manufacturing in a convenient manner; (d) binder-free; and (e) no additional drying process [23,24].

In the present study, the NPDS was used to fabricate ZnO–WO_3_ hybrid photoanodes at different WO_3_ contents (5, 10, 15, and 20 wt.%) on fluorine-doped tin oxide (FTO) substrates. The deposition process involved the localized and spontaneous fragmentation of microsized powder into nanosized thin films at room temperature and low vacuum conditions. The modified working electrodes with ZnO–WO_3_ hybrid NCs were utilized as photoanodes for the PEC water-splitting process in a neutral medium. Our study systematically explored the impact of varying tungsten oxide (WO_3_) weight ratios on charge transfer kinetics, bandgap engineering, and the efficiency of photogenerated charge carrier separation. The present study research outcomes demonstrate the composition-dependent PEC performance of ZnO–WO_3_ hybrid photoanodes, which need to be optimized to improve the overall performance for real-life applications.

## 2. Experimental

### 2.1. Material Details

Microsized ZnO powder (<5 µm particle size, 99.9%, CAS # 1314–13–2, Sigma–Aldrich, St. Louis, MO, USA) and WO_3_ microsized powder (≤25 µm, ≥99% trace metals basis, CAS # 1314–35–8, Sigma–Aldrich, St. Louis, MO, USA) were used to fabricate ZnO–WO_3_ nanocomposites on a 2.3 mm thick FTO (100 × 100 mm^2^, surface resistivity = 7 Ω/sq, Sigma–Aldrich, St. Louis, MO, USA). A 0.5 M Na_2_SO_4_ solution electrolyte was used as an agent with a pH of 6.8 (CAS# 7757–83–7, Duksan, Ansan-si, Republic of Korea) for the investigation of PEC water splitting.

### 2.2. Dry Coating of ZnO–WO_3_ Thin Films by the NPDS

**Figure 1:**
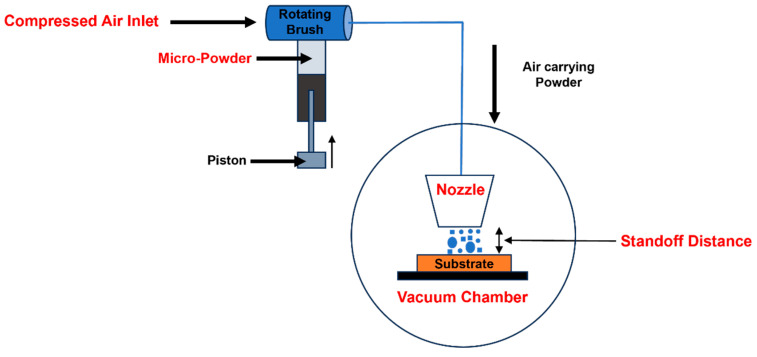
Deposition of nanostructured zinc–oxide–tungsten oxide hybrid photoanodes using the nanoparticle deposition system.

Nanostructured ZnO–WO_3_ thin films with varying WO_3_ (5%, 10%, 15%, and 20%) were deposited on the FTO by a one-step vacuum kinetic spray process, the NPDS. The NPDS is composed of an air compressor that supplies high pressure, a cylindrical piston known as a powder feeder, a vacuum pump, a nozzle, a controller, and a pressure chamber. The pressure was adjusted through the controller, and the microsized dry powder was mixed using a ball mill filled with a powder feeder that moved the powder to the powder chamber, where a rotating brush drives the powder and high-speed air pressure from the air compressor enters the nozzle. The powder was sprayed onto the substrate using a converging–diverging nozzle at room temperature. The impact of high-speed powder particles and the incident angle of the FTO substrate fractured the microsized powder into a nanosize range [25]. The conditions for deposition were set to a 3 mm standoff distance. The air compressor pressure was 3 bar, and the chamber pressure was 0.50 bar. More details are shown in Figure 1. The fabrication time for 40 × 10 mm^2^ samples using simple scanning and vacuum was less than 30 min. After deposition, there was no post-processing.

### 2.3. Photoelectrochemical Water Splitting Characteristics

An electrochemical workstation (Model: C350, Wuhan Corr-Test Instruments Corp. Ltd., Wuhan, China) and a solar stimulator (Model: 10500, ABET Technologies, Milford, CT, USA) with a 150 W Xe Arc Lamp as a light source were used in a three-electrode setup to evaluate ZnO–WO_3_ NCs in a 0.5 M Na_2_SO_4_ electrolytic solution. ZnO/FTO, WO_3_/FTO, and ZnO/WO_3_/FTO hybrid photoanodes with varying WO_3_ content were used as the working electrodes. The counter electrode was platinum (Pt) with a mesh area of 1 × 1 cm^2^, and the reference electrode was Hg/HgO in a 0.5 M Na_2_SO_4_ electrolyte solution. Linear sweep voltammetry (LSV) profiles were examined within the potential range of −0.499–1.001 vs. Hg/HgO at a scan rate of 10 mV·s^−1^. The potential range vs. the reference electrode was then converted to the reversible hydrogen electrode (RHE) scale using Equation (1) [26].

(1) $$E_{RHE}={E^{O}}_{Hg/HgO}+E_{Hg/HgO}+0.059\times pH$$

where ${E^{O}}_{Hg/HgO}$ is the reference electrode potential vs. SHE (0.059), the pH is 6.8, and $E_{Hg/HgO}$ is the applied potential. Therefore, the LSV curves were converted to 0–1.5 vs. the RHE. The charge transfer resistance (R_ct_) was evaluated by Nyquist plot under illumination with an AC frequency of 10 mV and a frequency range of 1 MHz–0.001 Hz. Photocurrent stability was assessed through a series of chronoamperometric (CA) measurements at a DC potential of 1.23 V vs. the RHE over five full cycles under both dark and light conditions. Mott–Schottky (M–S) plots were created in the dark using electrochemical impedance spectroscopy with an AC signal of 10 mV ranging from −1–0 V vs. Hg/HgO at a frequency of 1 kHz.

### 2.4. Material Characterization

The surface morphology was examined using scanning electron microscopy (SEM, Model: S-4800, Hitachi, Chiyoda City, Tokyo, Japan) of micropowder and nanocomposite thin films. The structures of ZnO, WO_3_, and hybrid photoanodes thin films and powders were examined using X-ray diffraction (XRD, Smart Lab, Shibuya-ku, Tokyo, Japan) with the X-ray wavelength Cu K-alpha from a diffraction angle of 10° to 90°. The Raman spectra for micropowder and nanostructured thin films were examined using a 1 mW laser source with a 532 nm excitation wavelength (Model: Alpha 300R, WITec, Ulm, Germany) in the range of 200–1200 cm^−1^. Furthermore, the bonding states within ZnO, WO_3_, and ZnO with varying WO_3_ content photoanodes binding energies ranging from 0 to 1200 eV were examined using a source K-alpha X-ray photoelectron spectrometer. Photoluminescence (PL) emissions were recorded using a fluorescence spectrometer (Cary Eclipse, Varian, Santa Clara, CA, USA) in a range of 350–600 nm wavelength. Furthermore, the optical diffuse reflectance (DRS) of nanostructured ZnO, WO_3_, and ZnO–WO_3_ hybrid photoanodes was measured using a UV–Vis–NIR spectrophotometer (Cary 5000, Agilent, Santa Clara, CA, USA) in the wavelength range of 300–699 nm.

## 3. Results and Discussion

### 3.1. XRD Analysis

**Figure 2:**
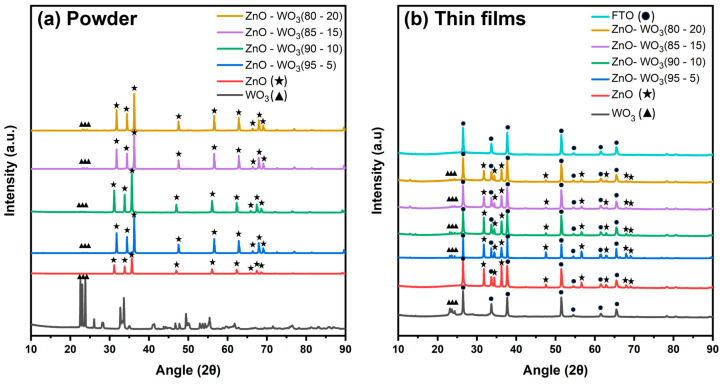
X-ray diffraction patterns of ZnO–WO_3_ in powder form (**a**) and ZnO–WO_3_ coated on a fluorine-doped tin oxide substrate (**b**).

The crystalline structures of ZnO, WO_3_, and ZnO–WO_3_ mixed micron particle powders at various concentrations of 5, 10, 15, and 20 wt.% WO_3_ were examined using XRD patterns (Figure 2a). The XRD pattern of ZnO shows characteristic diffraction peaks at 31.16°, 33.82°, 35.64°, 46.96°, 56.02°, 62.3°, 65.82°, 67.42°, and 68.56°. These peaks correspond to (1 0 0), (0 0 2), (1 0 1), (1 0 2), (1 1 0), (1 0 3), (2 0 0), (1 1 2), and (2 0 1) hexagonal ZnO phase crystalline planes (space group: P6_3mc_, 01–086–8198), respectively [27]. The microsized WO_3_ powder exhibited three main diffraction peaks at 22.56°, 23.04°, and 23.82°, corresponding to (0 0 2), (0 2 0), and (2 0 0) monoclinic WO_3_ phase crystalline planes (space group: P2_1/n_, 00–043–1035), respectively [28]. The composite powders, composed of various proportions of ZnO with WO_3_ content ranging from 5% to 20%, displayed peaks corresponding to both ZnO and WO_3_. This observation suggests the coexistence of hexagonal ZnO and monoclinic WO_3_ in the composite materials. Additionally, there is no new hybrid structural phase because of the predeposition grinding achieved through the ball milling process [29].

Figure 2b shows the XRD patterns of the bare FTO substrate and nanostructured thin films of ZnO, WO_3_, and ZnO–WO_3_ hybrid NCs at different WO_3_ contents (5, 10, 15, and 20 wt.%) on FTO. The XRD pattern of FTO shows several peaks at 26.48°, 33.66°, 37.72°, 54.5°, 61.5°, and 65.46°. The XRD pattern of the nanosized pure ZnO thin film shows peaks at 31.78°, 34.42°, 36.36°, 47.56°, 56.58°, 62.86°, 67.94°, and 69.08°. For the WO_3_ thin film, only major peaks are visible at 23.12°, 23.64°, and 24.38° because of its low content in the nanocomposites (NCs). It is observed that the thin film peak positions have shifted a little compared to the powder peak positions. This is because of fracturing, shattering, and random ordering of the particles during deposition [30]. Overall, the XRD patterns of the ZnO, WO_3_, and ZnO–WO_3_ NC thin films show relatively smaller peak intensities than those of the corresponding powder. This behavior is attributed to the polycrystalline nature of the nanostructured films deposited by the NPDS under rough vacuum conditions during the kinetic spray process [31]. This demonstrates the fragmentation of the initial microsized particles into many domains with a small nanosize, which is accompanied by an increase in the number of defective sites. These defect states act as additional reaction sites for oxygen evolution [23].

### 3.2. Raman Spectra Analysis

**Figure 3:**
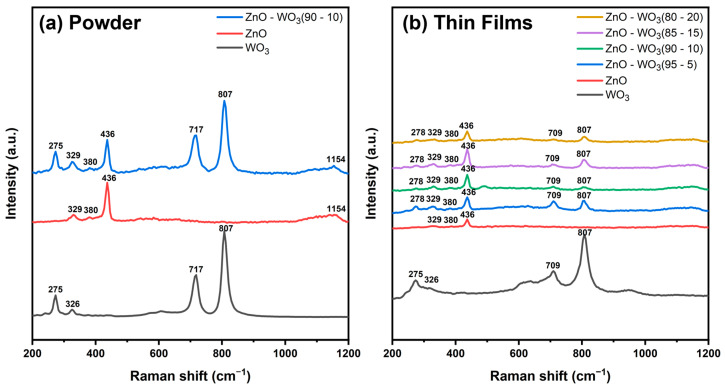
Raman spectra of WO_3_, ZnO, and ZnO–WO_3_ (10%) powders (**a**) and WO_3_, ZnO, and ZnO–WO_3_ (5%, 10%, 15%, and 20%) thin films on fluorine-doped tin oxide (**b**).

Raman analysis was used to confirm the crystal structure, crystal quality, and presence of both ZnO and WO_3_ species in the mixed micron powder and nanostructured thin films. The Raman spectrum of the WO_3_ micron powder shown in Figure 3a reveals several distinct peaks at 275, 326, 717, and 809 cm^−1^ [32]. Raman peaks at 275 and 326 cm^−1^ correspond to the stretching and bending vibrations of the lattice oxygenated bonds (W–O), respectively [33]. Meanwhile, the observed Raman peaks at higher Raman shift values of 717 and 807 cm^−1^ increase from several stretching vibrations related to W^VI+^–O of the monoclinic crystal structure, corresponding to the stretching vibrations of the bridging oxygen [34]. The prominent peaks detected at 807, 717, and 275 cm^−1^ exhibited distinct intensities and were characteristic vibrational modes of crystalline WO_3_ (monoclinic phase) [35,36,37]. The Raman spectrum of the ZnO powder in Figure 3a shows several distinct peaks at 329, 380, 436, and 1154 cm^−1^. The A1-symmetry mode at 380 cm^−1^ represents the first-order transverse optical (TO) phonon mode [38]. The E_2L_ mode corresponds to the low-frequency vibration of oxygen atoms, whereas the E_2H_ mode corresponds to the high-frequency vibration of heavy zinc (Zn) atoms. [39]. The prominent Raman mode at 329 cm^−1^ is primarily associated with the superimposition of second-order optical phonon vibration (E_2H_–E_2L_) [40,41]. The most prominent Raman peak at 436 cm^−1^ is related to the E_2H_ symmetry and nonpolar second-order vibration of the hexagonal crystalline structure of zinc oxide [42,43]. However, 1154 cm^−1^ represents the second-order vibration mode in ZnO. Similarly, the Raman spectrum for ZnO–WO_3_ (10%) mixed micron powder was observed, with all Raman peaks for WO_3_ powder (275, 717, and 809 cm^−1^) corresponding to the (W–O) lattice bond and stretching vibration related to W^VI+^–O (Figure 3a). In addition, the peak at 326 cm^−1^ of WO_3_ vanished in the ZnO–WO_3_ (10%) mixed micron powder because of its proximity to the ZnO powder peak at 329 cm^−1^. However, 380, 436, and 1154 cm^−1^ peaks of ZnO powder corresponding to the first-order TO phonon mode and superimposition of second-order optical phonon vibration (E_2H_–E_2L_) were present in the Raman spectra of the ZnO–WO_3_ (10%) powder (Figure 3a).

Figure 3b shows the Raman peaks for ZnO on the FTO substrate, indicating all peaks of the powder, which are 329, 380, and 436 cm^−1^, without any change in peak position, demonstrating that the hexagonal structure remained the same after deposition. The main peak, known as E_2_ (high) at 436 cm^−1^, is the fingerprint of the wurtzite crystal structures [44]. Peaks at 275, 326, and 807 cm^−1^ for WO_3_ nanostructure thin films show no change compared with powder peaks; however, the peak at 717 cm^−1^ shifted to 709 cm^−1^, indicating poor crystalline quality and fragmentation of WO_3_ particle size from micro to nano, as explained in the SEM analysis. Furthermore, ZnO–WO_3_ thin films on the FTO are shown in Figure 3b. All hybrid thin films showed the same peaks of ZnO and WO_3_. The observed peak at 275 cm^−1^ for WO_3_ showed a positive shift to 278 cm^−1^ for hybrid thin films, indicating an interaction between ZnO and WO_3_. However, the 717 cm^−1^ peak in the WO_3_ powder shifted negatively to 709 cm^−1^, indicating a decrease in the crystalline quality and overall fragmentation by lattice disorder, as explained in the XRD analysis.

### 3.3. SEM Analysis

The surface morphologies of ZnO, WO_3,_ and ZnO–WO_3_ with 10% WO_3_ powder were measured using SEM (Model: S-4800, Hitachi High-Technologies) (Figure S1). The shapes and sizes of the ZnO and WO_3_ particles were observed. The size of the WO_3_ microparticle powder ranges <50 µm. However, ZnO powder showed multiple variations in sheets and rods with particles ranging <25 µm. Similarly, the presence of both zinc oxide and tungsten oxide particles was visible in the ball-milled ZnO–WO_3_ (10%) mixture before deposition of similar shapes and sizes <25 µm.

**Figure 4:**
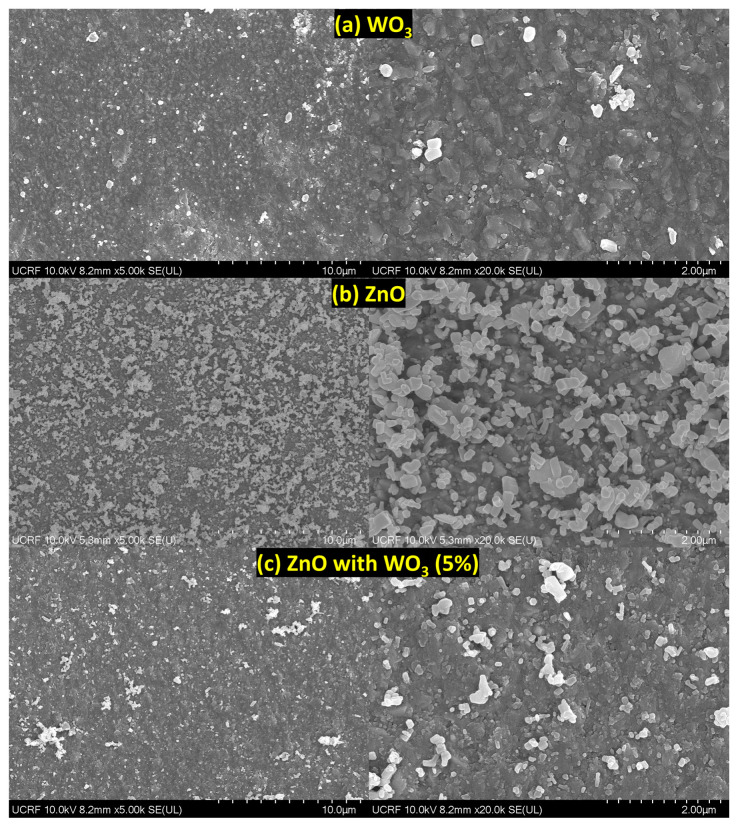
Scanning electron microscopy images of WO_3_ (**a**), ZnO (**b**), and ZnO with 5% WO_3_ (**c**) content on fluorine-doped tin oxide.

**Figure 5:**
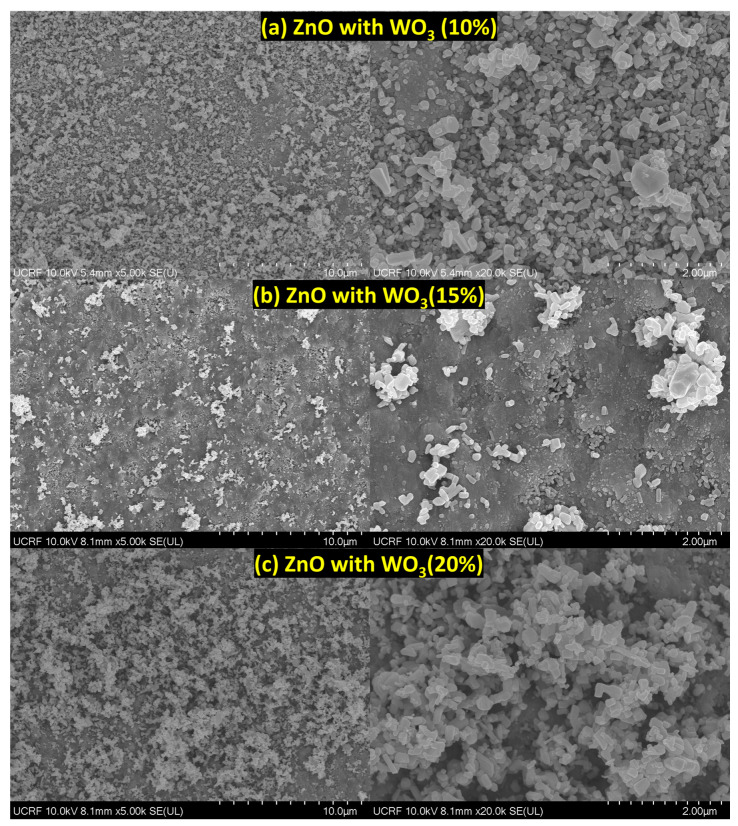
Scanning electron microscopy images of ZnO with 10% (**a**), ZnO with 15% (**b**), and ZnO with 20% (**c**) WO_3_ content on fluorine-doped tin oxide.

Figure 4a shows WO_3_ deposited on the FTO substrate; the particle size was significantly reduced from 25 µm to <500 nm, and the SEM image showed several nanosheets. This could be due to the dispersion of the grains in each particle after deposition because of high kinetic energy or the impact between accelerated microparticles and the substrate, causing microparticles to fracture to a smaller nanosize during deposition. When a ZnO thin film was deposited on FTO, multiple variations in nanosheets and nanorods with particle sizes ranging <500 nm were observed (Figure 4b). Therefore, large particles of ZnO and WO_3_ were fragmented into smaller particles (particularly of grain size before deposition) because of the accelerated particles with high-impact energy collision with the metallic substrate (FTO) (Figure S1a,b). The SEM morphologies of the hybrid ZnO–WO_3_ (5%, 10%, 15%, and 20%) thin films are shown in Figure 4c and Figure 5a–c. All hybrid nanocomposites contain particles of similar shape and size (<500 nm). An overall uniform distribution of particles is observed in the microscale micrographs of all thin films. The shift from microscale powder to nanosized particles in all NC thin films resulted in more defective sites, which acted as more reaction sites, as explained in the XRD analysis.

### 3.4. XPS Analysis of ZnO–WO_3_ NCs

**Figure 6:**
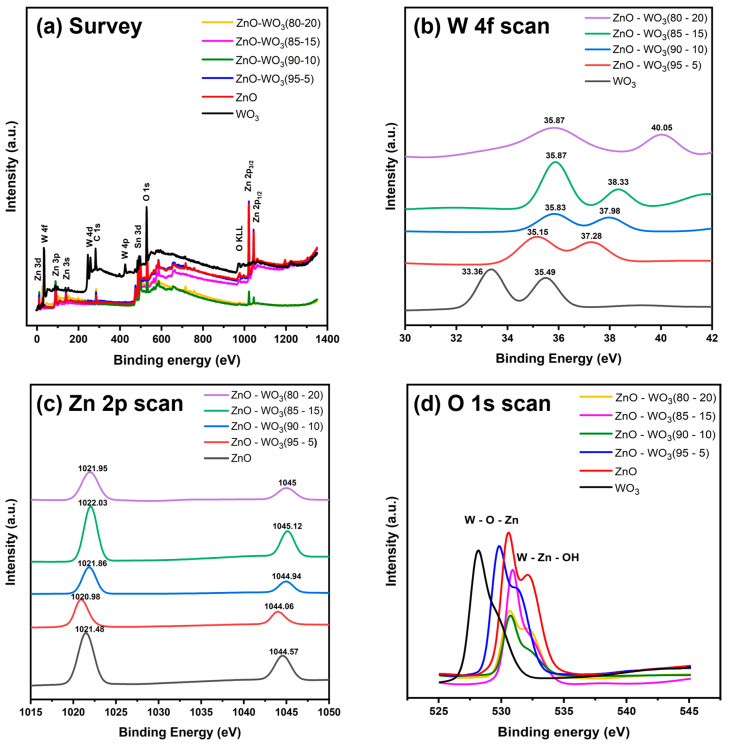
X-ray photoelectron spectroscopy (XPS) survey spectrum (**a**), high-magnified W 4f (**b**), Zn 2p (**c**), and O 1s (**d**) XPS scans of ZnO–WO_3_ hybrid nanocomposites deposited on fluorine-doped tin oxide.

The interfacial chemical bonding states on ZnO NSs, WO_3_ NSs, and ZnO–WO_3_ hybrid NCs at different WO_3_ contents (5, 10, 15, and 20 wt.% WO_3_) were investigated using XPS survey spectra (Figure 6a), which revealed the presence of typical signals corresponding to Zn, W, C, Sn, and O elements. These signals were characterized by the active states of ZnO, WO_3_, and FTO from the holding substrate. The high-magnified XPS scans of W 4f, Zn 2p, and O 1s are illustrated in Figure 6b–d.

**Figure 7:**
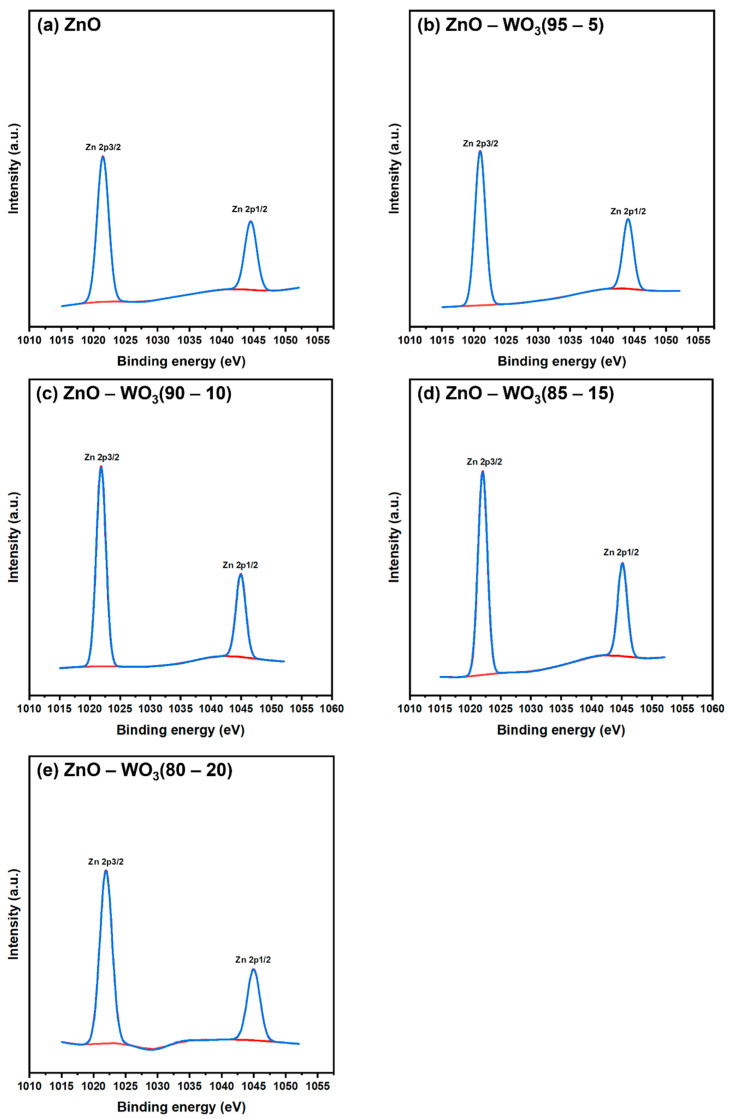
Deconvoluted Zn 2p scans of ZnO NSs (**a**) and ZnO–WO_3_ NCs with 5 wt.% (**b**), 10 wt.% (**c**), 15 wt.% (**d**), and 20 wt.% (**e**) WO_3_.

Figure 7a–e shows the deconvolute XPS scans of the Zn 2p band for both pure ZnO NSs and ZnO–WO_3_ hybrid NCs at various WO_3_ contents: 5 wt.%, 10 wt.%, 15 wt.%, and 20 wt.%. In the high-resolution Zn 2p XPS scan of pure ZnO NSs, two distinct subband peaks were observed, attributing to Zn 2p_3/2_ and Zn 2p_1/2_ at 1021.48 and 1044.57 eV, respectively. The estimated peaks in the ZnO–WO_3_ NCs at various WO_3_ contents are shown in Table 1. The average energy separation between Zn 2p_3/2_ and Zn 2p_1/2_ was approximately 23.09, which corresponds to the metallic Zn phases [45]. The binding energy of the deconvolute Zn 2p peaks negatively shifts with increasing WO_3_ content up to 5 wt.%; however, increasing WO_3_ contents (10 wt.%–20 wt.%) causes a positive shift in binding energy to a higher value than with ZnO NCs. This indicated that improving surface bonding and synergy between ZnO and WO_3_ in the hybrid NCs could enhance the interfacial electron density at ZnO grain boundaries (Table 1).

**Figure 8:**
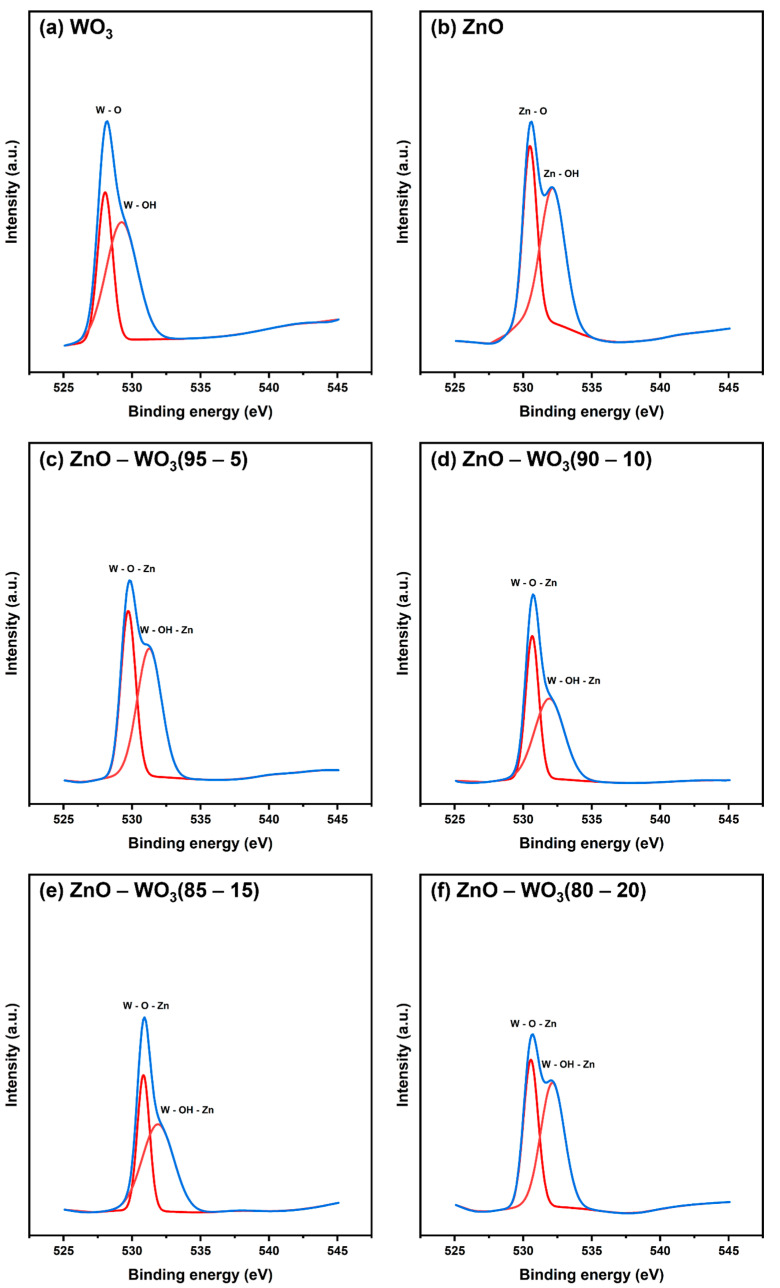
Deconvoluted O 1s X-ray photoelectron spectroscopy scans of WO_3_ NSs (**a**), ZnO NSs (**b**), and hybrid ZnO–WO_3_ NCs at 5 wt.% (**c**), 10 wt.% (**d**), 15 wt.% (**e**), and 20 wt.% (**f**) WO_3_.

Figure 8a shows high-resolution deconvoluted O 1s scans of WO_3_ NSs with double degenerate binding states at 530.49 and 532.16 eV [46,47]. These bands showed the presence of oxygenated bonds, including internal lattice oxygen (W–O) and adsorbed water molecules (W–OH) [48]. However, the characteristic oxygenated bonds shown in Figure 8b of ZnO were detected at 528.04 and 529.24 eV, which are attributed to Zn–O and Zn–OH, respectively [15,49]. The deconvolute O 1s scans of ZnO–WO_3_ NCs at various WO_3_ contents (5–20 wt.%) are illustrated in Figure 8c–f, and the estimated peak positions are shown in Table 1.

**Figure 9:**
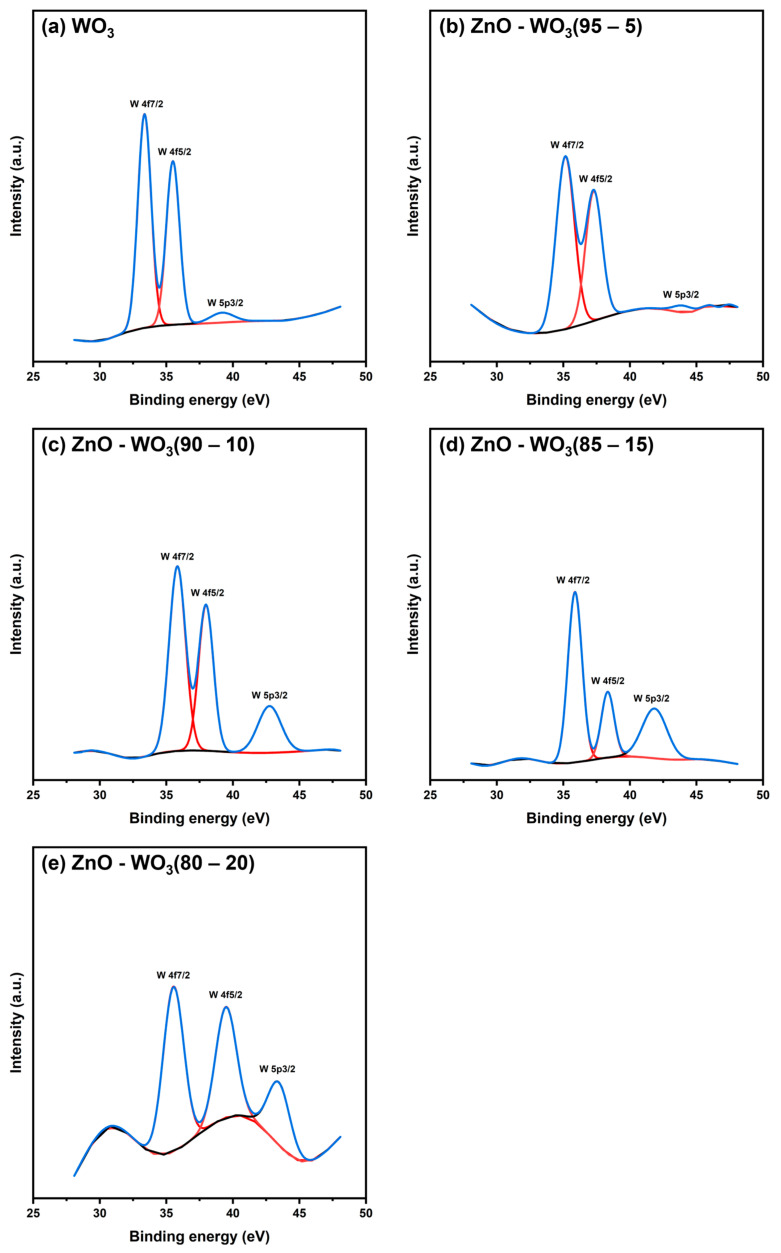
Deconvoluted W 4f X-ray photoelectron spectroscopy scan of WO_3_ (**a**), 5% WO_3_ (**b**), 10% WO_3_ (**c**), 15% WO_3_ (**d**), and 20% WO_3_ (**e**) heterostructure thin films.

Figure 9a shows deconvoluted W 4f XPS scans of pure WO_3_ NSs, which demonstrated the presence of W 4f_7/2_ at 33.36 eV and W 4f_5/2_ at 35.49 eV and a small peak of 5p_3/2_ in WO_3_ [50,51]. Furthermore, ZnO with WO_3_ (5%, 10%, 15%, and 20%) heterostructure thin films in all three main peaks are shown in Figure 9b–e, and their values are listed in Table 1. The heterostructure nanocomposites with different WO_3_ contents showed that increasing the WO_3_ content leads to a positive binding energy peak shift for both 4f_7/2_ and 4f_5/2_ peaks, demonstrating the strong coupling between WO_3_ and ZnO in the deposited heterostructure films within the nanoscale range. Additionally, this obvious positive shift can be due to the presence of interfacial charge transfer from WO_3_ to ZnO in the ZnO/WO_3_ heterojunction interface. When WO_3_ and ZnO are combined in different compositions, the formation of heterojunctions between the two semiconductors means that the WO_3_ semiconductor will transfer interfacial charge to the ZnO semiconductor through the formed interface, thus leading to an increase in W binding energy (Figure 6b) and a decrease in Zn binding energy [52]. More W content means more charge transfers. Furthermore, it is also visible in estimated valence band position section that the conduction band position of WO_3_ is −3.35 eV above ZnO at −0.65 eV.

### 3.5. Analysis of the Optical Bandgap of ZnO–WO_3_ Hybrid NCs

**Figure 10:**
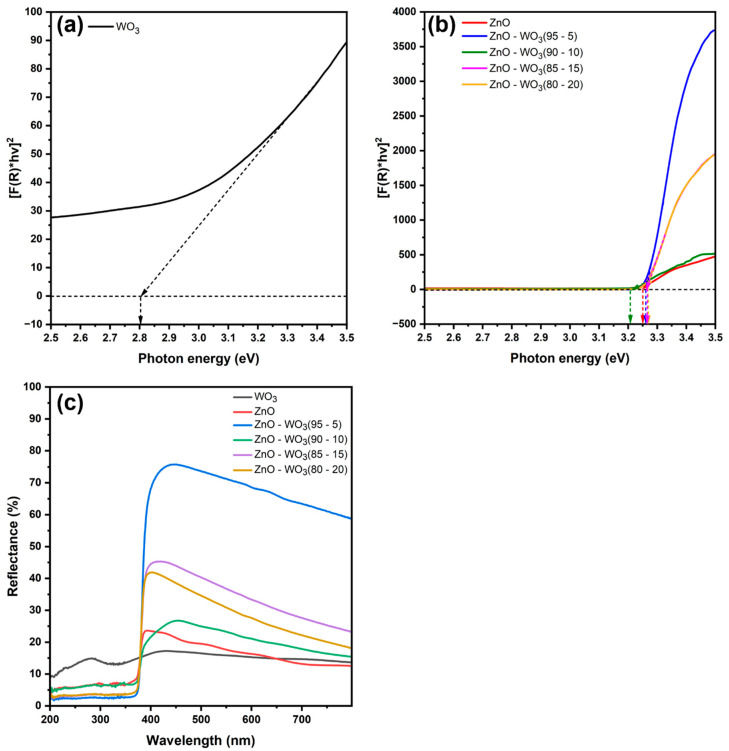
Calculated Tauc’s plot of WO_3_ (**a**), the calculated Tauc’s plot (**b**), and ultraviolet–visible diffuse reflectance spectra (**c**) of ZnO–WO_3_ hybrid nanocomposites photoanodes.

Diffuse reflectance (R%) spectroscopy was used to examine the optical properties of pure ZnO nanosheets, WO_3_ nanosheets, and ZnO–WO_3_ NC hybrid photoanodes at different weight ratios of WO_3_ (5 wt.%, 10 wt.%, 15 wt.%, and 20 wt.%) in the wavelength range of 200–799 nm (Figure 10a,b). Tauc’s plots of ZnO–WO_3_ NCs at different WO_3_ contents are shown in Figure 10a, where linear extrapolation in the high energy region provided the optical bandgap value of direct bandgap semiconductors according to Equation (2) [53,54].

(2) $$\frac{\alpha }{S}=F={\frac{\left(1-R\right)}{2\times R}}^{2}$$

(αhv)^2^ = C(hv − E_g_) (3)

where α is the absorption coefficient, v is the incident light frequency, E_g_ is the optical bandgap energy, h is the Planck constant, C is the proportionality constant, and *S* is the scattering coefficient.

The estimated E_g_ values of pure nanostructured thin films of WO_3_ and ZnO are 2.8 and 3.25 eV, respectively. The incorporation of WO_3_ into the hybrid NCs did not significantly affect the band structure of the ZnO host lattice, where the estimated E_g_ values in ZnO–WO_3_ hybrid NCs at WO_3_ ratios of 5, 10, 15, and 20 wt.% were 3.26, 3.20, 3.264, and 3.27 eV, respectively.

**Figure 11:**
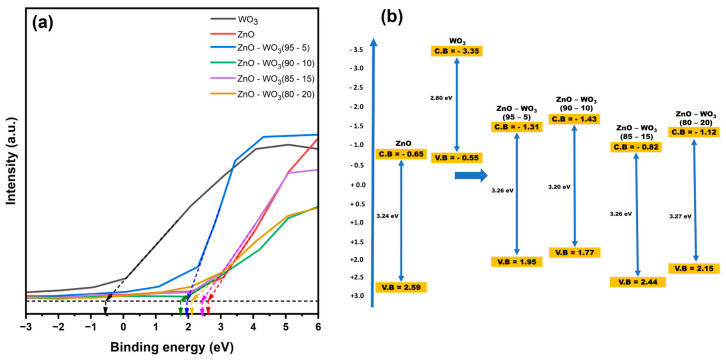
Valence band calculation from the survey spectra of ZnO, WO_3_, and ZnO–WO_3_ (5%, 10%, 15%, 20%) (**a**). Bandgap structure of all photoanodes (**b**).

Figure 11a shows the valence band position construction using the XPS survey spectra for binding energies ranging from −3 to 6 eV. The estimated valence band position of ZnO nanosheets is 2.59 eV, that of WO_3_ nanosheets is −0.55 eV, and that of ZnO–WO_3_ hybrid NCs at different WO_3_ contents of 5, 10, 15, and 20 wt.% are 1.95, 1.77, 2.44, and 2.15 eV, respectively (Figure 11b). When energy is absorbed that exceeds the bandgap of the semiconductor, electrons in the valence band (VB) are excited, whereas holes remain in the VB. These holes participate in water oxidation to produce O_2_. To facilitate water splitting, the VB potential must exhibit a more positive value than the redox potential of O_2_/H_2_O, which is equivalent to 1.23 eV. The incorporation of WO_3_ in ZnO negatively shifts the valence position in the ZnO–WO_3_ hybrid NCs.

### 3.6. Photoluminescence Emission Behavior of ZnO–WO_3_ Hybrid NCs

**Figure 12:**
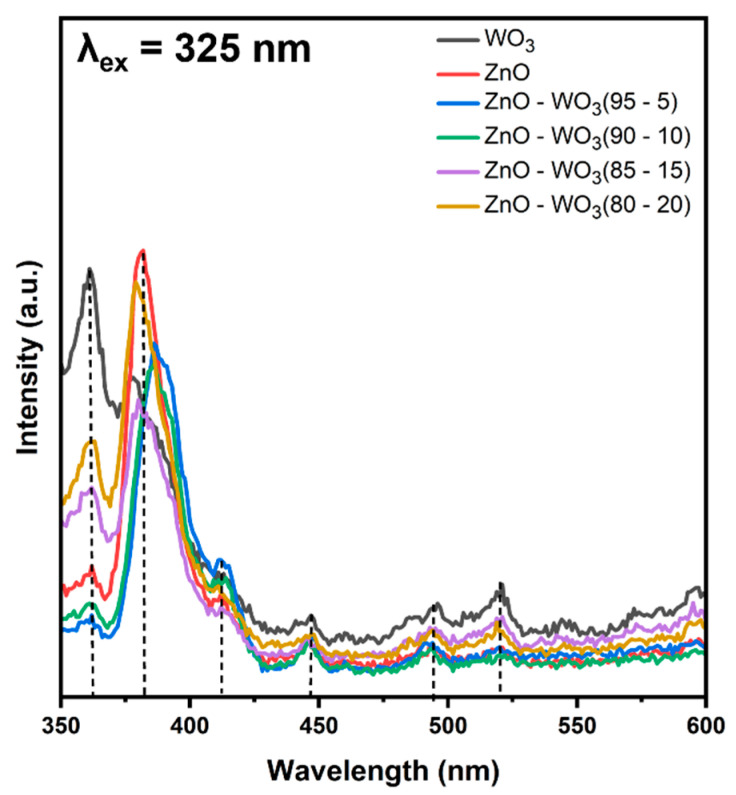
Photoluminescence emission spectra of ZnO NSs, WO_3_ NSs, and ZnO–WO_3_ hybrid nanocomposites at various WO_3_ content.

Photoluminescence (PL) emission spectroscopy is more sensitive than normal optical absorbance to localized states induced by interfacial structural defects. The depth of these states within the forbidden gap can be determined by observing defect-related PL spectra arising from the recombination of trapped charge carriers with photogenerated holes [55]. In this study, the effect of interfacial hybridization between WO_3_ and ZnO species in ZnO–WO_3_ NCs at different WO_3_ contents was monitored using PL emission spectra (Figure 12).

The PL emission spectrum of ZnO NSs in Figure 12 at an excitation wavelength of 325 nm exhibited broad emission in the UV–visible spectral region [56], with observed emission peaks at 362, 382, 411, 445, 494, and 520 nm. The UV emission bands at 362 and 382 nm were ascribed to fundamental band-edge emission and excitonic recombination [55,57]. The PL emission in the violet–blue spectra region at 411 and 445 was attributed to the trapped electrons recombining with photogenerated holes. These trapped electrons are located at shallow trapping levels, where they are linked to either interstitial zinc or oxygen vacancies [58]. The recombination of trapped electrons at deep trapping sites of single oxygen vacancies with photogenerated holes was mainly due to green emission around 520 nm [59,60,61]. Although the estimated optical bandgap of WO_3_ NSs was lower than that of pure ZnO NSs (Figure 10a,b), similar emission behavior was observed. The most intense UV emission band of the WO_3_ NSs was observed at a lower wavelength of 362 nm than that of the ZnO NSs (378 nm). However, other emission bands were observed in the visible spectral range, covering violet (411 nm), blue (453 and 487 nm), and green (523 nm) emission bands [62]. The emission bands observed in the blue–green spectral region (453 and 487 nm) lie in the same energy range as the WO_3_ bandgap (~2.8 eV). Therefore, they could be attributed to fundamental interband transitions [63,64,65]. The green emission observed at 523 nm occurred at an energy lower than the bandgap of WO_3,_ which is ascribed to the recombination of trapped electrons in deep-localized oxygen vacancies with photogenerated holes [66]. Similar emission bands were observed in ZnO–WO_3_ hybrid NCs at different WO_3_ contents (5 wt.%, 10 wt.%, 15 wt.%, and 20 wt.%). Generally, the hybridization between ZnO and WO_3_ led to a decrease in the PL emission intensity compared with the pure phases of ZnO and WO_3,_ indicating quenching of the photogenerated carrier recombination rates. This behavior indicates an improvement in synergy in hybrid NCs, which is accompanied by an improvement in photon energy conversion efficiency [15,67,68].

### 3.7. Photoelectrochemical Water Splitting Measurements

**Figure 13:**
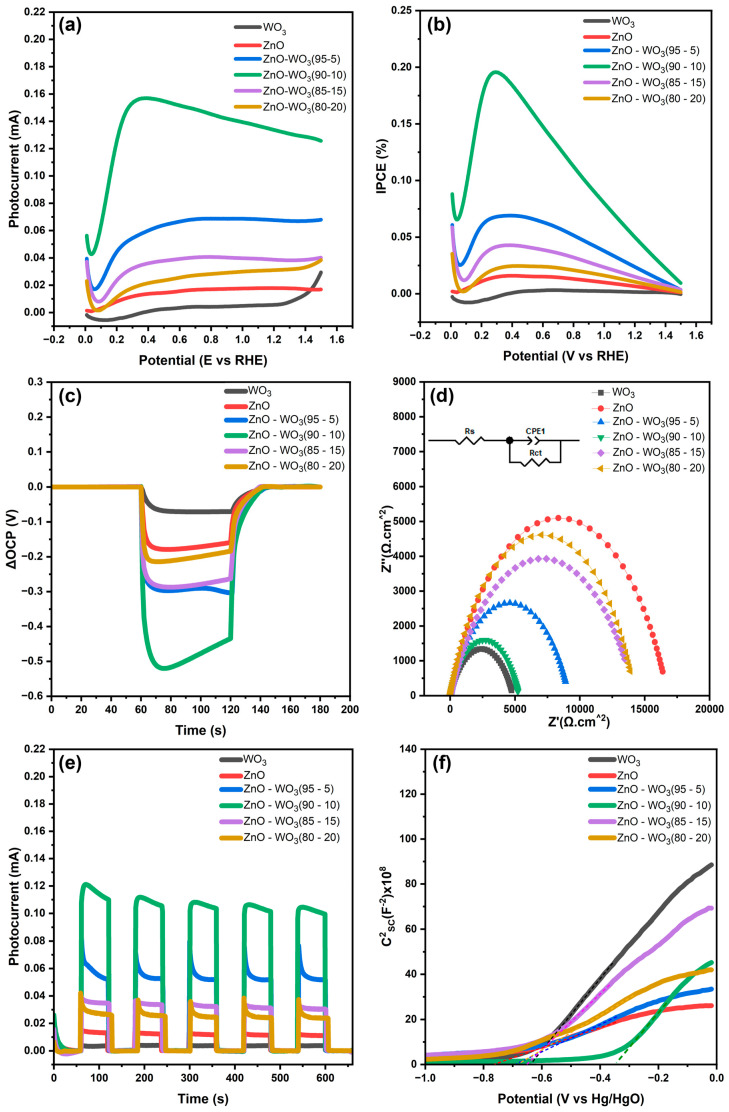
Photoresponse current (**a**), incident photon conversion efficiency (**b**), the difference in open-circuit potential (**c**), Nyquist plots under illumination (**d**), and chopped chronopotentiometry (**e**) at 1.23 V vs. the RHE, and M–S plots (**f**) of ZnO–WO_3_ NC photoanodes at various WO_3_ %.

PEC water oxidation measurements were performed in a neutral medium of 0.5 M Na_2_SO_4_ for a modified FTO working electrode with ZnO nanosheets, WO_3_ nanosheets, and a ZnO–WO_3_ hybrid (NCs at various WO_3_ contents of 5 wt.%, 10 wt.%, 15 wt.%, and 20 wt.%). Figure 13a shows the photocurrent response of all modified photoanodes in the potential range of 0–1.5 V vs. the RHE (i.e., from −0.5 to 1 vs. Hg/HgO). The measured photocurrent ZnO–WO_3_ NC hybrid photoanodes with WO_3_ weight ratios of 5, 10, 15, and 20 wt.% were 66, 133, 38, and 31 μA, respectively. The obtained photoresponse current of hybrid ZnO–WO_3_ NCs was higher than that of pure nanostructured phases of ZnO (18 μA) and WO_3_ nanosheets (6 μA), demonstrating the improvement of photocurrent response compared with the pure phases as a result of the improved synergy between ZnO and WO_3_ species in the hybrid NCs, which was accompanied by the enhancement of interfacial charge transfer at the electrode/electrolyte interface. The incident photon-to-current efficiency (IPCE) of the fabricated hybrid photoanodes at different polarization potentials was determined using Equation (4) [69]:

(4) $$IPCE\left(\%\right)=J_{ph}\frac{\left(1.23-(V-V_{OCP}\right)}{P_{light}}\times 100\%$$

where *P_light_* is the incident power light density, *J_ph_* is the photocurrent density, *V* is the polarization potential, and *V_OCP_* is the change in the open-circuit potential (ΔOCP) under light (Figure 13c). Achieving a higher open-circuit potential is crucial in PEC water splitting because it determines the maximum thermodynamic efficiency of the overall water-splitting process. In this study, ZnO–WO_3_ NCs with a 10 wt.% WO_3_ hybrid photoanode achieved the highest open-circuit potential (OCP). Higher open-circuit potentials are important for optimal PEC cell operation [70,71]. Figure 13b shows the potential-dependent IPCE (%), which is the calculated IPCE (%) value at 1.23 V vs. the RHE (Table 2).

Figure 13d shows Nyquist plots of ZnO NSs, WO_3_ NSs, and ZnO–WO_3_ NC hybrid photoanodes under sunlight illumination at 1.23 V vs. the RHE. The incorporation of WO_3_ resulted in a reduction in the charge transfer resistance (R_ct_) of ZnO–WO_3_ hybrid NCs compared with that of pure ZnO NSs, indicating an enhancement in the kinetics of interfacial charge transfer. To further assess the photoelectrochemical (PEC) capabilities, the Electrical Equivalent Circuit (EEC) model was analyzed for the fabricated photoanodes while being illuminated at 1.23 V_RHE_ (Figure 13d). The accompanying inset illustrates an RC circuit fitted to the data, with R_s_ representing solution resistance, R_ct_ denoting interfacial charge transfer resistance between the photoanode and electrolyte interfaces, and CPE as a constant phase element. Table 3 provides the calculated values of R_ct_, R_s_, and CPE based on the equivalent circuit scheme. The consistent R_s_ values of 10 Ω (± 4 Ω) across all samples reflect stable solution conditions. However, the observed R_ct_ value of ZnO–WO_3_ (90–10) (5270 Ω) is markedly lower compared to ZnO and other nanocomposites. This lower R_ct_ for the ZnO–WO_3_ (90–10) interface indicates efficient separation of photogenerated electrons and holes in the photoanodes, attributed to the effective consumption of holes in the electrolyte solution. Furthermore, the semicircle diameter in the Nyquist plots represents the charge transfer behavior at the interfaces between the photoanodes and electrolytes. A smaller diameter signifies lower charge transfer resistance, indicating enhanced efficiency in the separation of photogenerated charge carriers [22,72]. Similarly, the stability of the photoresponse current was validated for five complete cycles under dark and illumination states for all, as shown, and hybrid photoanodes in Figure 13e.

Figure 13f shows Mott–Schottky plots of ZnO NSs, WO_3_ NSs, and ZnO–WO_3_ NCs at various WO_3_ contents (5%, 10%, 15%, and 20%), where the flat band potential (*V_fb_*) and the corresponding donor density concentration (*N_D_*) were estimated and recorded in Table 4.

(5) $$\frac{1}{{C_{Sc}}^{2}}=\frac{1}{2\epsilon \epsilon_{o}eA^{2}N_{D}}(V-V_{fb}-\frac{k_{B}T}{e})$$

where *A* is the electrode area, *V* is the applied potential, *e* is the electric charge of an electron, *T* is the absolute temperature, *k_B_* is the Boltzmann constant, ϵ is the dielectric constant of ZnO (8.6) [15] and WO_3_ (50) [73,74,75], and $\epsilon_{o}$ is the vacuum electric permittivity.

The combination of WO_3_ with ZnO nanosheets increased the positive shift of *V_fb_* for all hybrid photoanodes compared with photoanodes composed solely of pure ZnO NSs. Compared to all hybrid photoanodes, the hybrid ZnO–WO_3_ NCs containing 10 wt.% WO_3_ exhibited the highest positive shift. The observed positive shift in *V_fb_* indicates improved energy conversion efficiency [76]. However, the concentration of majority carriers (*N_D_*) in n-type semiconductors by a direct route does not correlate with PEC cell performance. This is because *N_D_* represents the concentration of charge carriers that do not participate in the PEC water-splitting reaction that occurs at the interface between the electrode and electrolyte. Despite this, alterations in *N_D_* exert a substantial influence on the positioning of the Fermi level (*E_f_*) concerning the intrinsic energy level (*E_i_*). This relationship is particularly relevant because (*E_i_*) is situated at the center of the modified band structure of the electrode according to the following relationship:

(6) $$E_{f}=E_{i}+kT\times ln(\frac{N_{D}}{n_{i}})$$

Furthermore, the concentration of minority carriers (holes) within the space charge layer contributes to water oxidation and O_2_ evolution during PEC water splitting. The space charge layer formed between the electrode and electrolyte plays a significant role in the performance of energy conversion. According to K. Schwarzburg [77], the separation of photogenerated electron-hole pairs occurs rapidly within picoseconds in the depletion region because of an externally applied potential. In this process, electrons generated by light absorption relocate to the interior bulk area of the photoanode surface, while holes created in the process traverse toward the position between the electrode and electrolyte, contributing to the PEC water splitting process [78]. W_scl_ can be calculated using Equation (7):

(7) $$W_{scl}=\sqrt{\frac{2\epsilon \epsilon_{o}(V-V_{fb})\;}{eN_{D}}}$$

The calculated values for W_scl_ are shown in Table 4. Based on the calculated W_scl_ values, the photoanode ZnO–WO_3_ (90–10) showed the highest value compared with the other hybrid photoanodes and pure ZnO/FTO. This explains why the incorporation of WO_3_ content in ZnO at a certain limit (10%) enhances the charge kinetics of ZnO–WO_3_ hybrid photoanodes and the concentration of photogenerated carriers within the space charge layer located at the interface between the electrode and electrolyte.

## 4. Conclusions

ZnO–WO_3_ NC hybrid photoanodes were deposited on an FTO substrate using a one-step dry NPDS with no additional post-process. The fabricated heterostructure electrodes were used to examine the PEC water splitting in a neutral electrolyte (0.5 M Na_2_SO_4_); SEM images clearly showed the microparticle transformation of nanosized structures in the deposited thin films. Raman spectra revealed a decrease in crystallinity due to kinetic-induced fragmentation in all hybrid photoanodes at WO_3_ contents ranging from 5 to 20 wt.%. High-resolution XPS of the W 4f, Zn 2p, and O 1s bands revealed a negative shift with increasing ZnO content in the hybrid NCs, demonstrating improved interfacial synergy. Analysis of the diffuse reflectance spectra demonstrated that increasing the WO_3_ to 10 wt.% reduced the ZnO bandgap from 3.24 to 3.20 eV in the hybrid photoanode. The PL emission spectra revealed that the ZnO with 10 wt.% WO_3_ hybrid photoanodes showed the lowest emission intensity, indicating that the dissociation of photogenerated charges was improved. Analysis of Mott–Schottky plots across all hybrid photoanodes indicated a positive shift in the V_fb_, reduction in N_D_, and expansion of the space charge layer width compared with ZnO/FTO. This phenomenon exerted a substantial impact on the effective segregation of photogenerated electron-hole pairs within the space charge layer located at the interface between the electrode and electrolyte, leading to an overall enhancement in PEC water splitting. Note that the introduction of WO_3_ into all hybrid heterostructure electrodes increased the photoresponse current and reduced the charge transfer resistance compared with nanoscale ZnO/FTO photoanodes. The ZnO–WO_3_ NCs/FTO hybrid photoanodes with 5%, 10%, 15%, and 20% WO_3_ content exhibited photocurrents of 0.066, 0.133, 0.038, and 0.031 mA·cm^−2^, respectively, compared with only 0.018 mA·cm^−2^ for pure ZnO/FTO and 0.006 mA·cm^−2^ for WO_3_/FTO photoanodes at 1.23 V vs. the RHE. Furthermore, the maximum IPCE for ZnO–WO_3_ hybrid photoanodes exhibited a transition to a lower potential than that for ZnO/FTO and WO_3_/FTO photoanodes. The ZnO–WO_3_ hybrid photoanode with 10% WO_3_ content revealed a maximum efficiency of 0.196%. The stability of the photocurrent for all NS photoanodes was validated for five cycles. The stability test revealed almost the same photocurrents. In contrast to alternative methods for fabricating hybrid ZnO–WO_3_ nanocomposite photoanodes, the NC photoanodes in this study were fabricated in a very short time (Table S1) using only the one-step dry NPDS method with commercially available microsized powders mixed with conventional ball milling. In addition, no additional post-processes for binding, cleaning, or drying were required. Finally, a 10% ZnO–WO_3_ photoanode outperformed pure ZnO/FTO, WO_3_/FTO, and other NC photoanodes in terms of PEC water splitting in a neutral electrolyte.

## Figures and Tables

**Table 1:** Band centers deconvoluted Zn 2p, O 1s, and W 4f X-ray photoelectron spectroscopy scans of ZnO–WO_3_ nanocomposite hybrid photoanodes at various WO_3_ contents (wt.%).

Band	Binding Energy (eV)					
	WO_3_	ZnO–WO_3_ (80–20%)	ZnO–WO_3_ (85–15%)	ZnO–WO_3_ (90–10%)	ZnO–WO_3_ (95–5%)	ZnO
W 4f	33.36	35.87	35.87	35.83	35.15	-
	35.49	40.05	38.33	37.98	37.28	-
O 1s	528.04	530.56	530.83	530.66	529.72	530.49
	529.24	532.14	531.90	531.90	531.27	532.16
Zn 2p	-	1021.95	1022.03	1021.86	1020.98	1021.48
	-	1045	1045.12	1044.94	1044.06	1044.57

**Table 2:** Photoresponse current (J_ph_) and photon conversion efficiency (IPCE) of ZnO–WO_3_ NC hybrid photoanodes at different WO_3_ contents.

Electrodes	Photocurrent (mA·cm^−2^)	IPCE (%)
WO_3_	0.006	0.003
ZnO	0.018	0.016
ZnO–WO_3_ NCs (5 wt.% WO_3_)	0.066	0.069
ZnO–WO_3_ NCs (10 wt.% WO_3_)	0.133	0.196
ZnO–WO_3_ NCs (15 wt.% WO_3_)	0.038	0.043
ZnO–WO_3_ NCs (20 wt.% WO_3_)	0.031	0.025

**Table 3:** Equivalent-circuit fitting parameters including nussiance resistance (R_s_), charge transfer resistance (R_ct_), and constant phase element (CPE) capacitance of ZnO–WO_3_ NC hybrid photoanodes at different WO_3_ contents.

Electrodes	R_s_ (Ω)	R_ct_ (kΩ)	CPE (F)
WO_3_	8.34	4.82	0.00021
ZnO	10.97	16.76	0.000099
ZnO–WO_3_ NCs (5 wt.% WO_3_) NCs	9.76	9.13	0.00017
ZnO–WO_3_ NCs (10 wt.% WO_3_) NCs	11.43	5.27	0.00016
ZnO–WO_3_ NCs (15 wt.% WO_3_) NCs	8.88	14.22	0.00017823
ZnO–WO_3_ NCs (20 wt.% WO_3_) NCs	13.09	14.56	0.00016

**Table 4:** Calculated donor concentration (*N_D_*), flat band potential (*V_fb_*), and width of the space charge layer (W_scl_) of hybrid ZnO–WO_3_ nanocomposite photoanodes across varying WO_3_ content levels.

Electrode	*V_fb_*	*N_D_* × 10^26^	W_scl_ (nm)
WO_3_	−0.64	0.46	12.84
ZnO	−0.78	9.24	1.24
ZnO–WO_3_ NCs (5 wt.% WO_3_)	−0.69	6.70	1.42
ZnO–WO_3_ NCs (10 wt.% WO_3_)	−0.36	2.69	1.96
ZnO–WO_3_ NCs (15 wt.% WO_3_)	−0.65	4.06	1.79
ZnO–WO_3_ NCs (20 wt.% WO_3_)	−0.69	5.70	1.53

## Data Availability

Data are contained within the article and Supplementary Materials.

## Supplementary Materials

The following supporting information can be downloaded at: https://www.mdpi.com/article/10.3390/mi14122189/s1, Figure S1: Scanning electron microscopy images of ZnO powder (a), WO_3_ powder (b), and ZnO–WO_3_ composite powder with 10% WO_3_ content (c). Table S1. The average consumable time for different material coatings in different techniques. Refs. [79,80,81] are cited in the Supplementary Materials.
