# Supplementary material for: One-Step Dry Coating of Hybrid ZnO–WO3 Nanosheet Photoanodes for Photoelectrochemical Water Splitting with Composition-Dependent Performance
Source: Micromachines (Basel). 2023 Nov 30;14(12):2189. doi: 10.3390/mi14122189 (PMC10745309; doi:10.3390/mi14122189)
Supplement: Supplementary file 1 [file micromachines-14-02189-s001.zip › micromachines-2731498-supplementary.pdf]

Supporting Information

One-step dry coating of hybrid ZnO-WO_3 nanosheet photoanodes for photoelectrochemical water splitting with composition-dependent performance

Muhammad Shehroze Malik [a], Deepto Roy [a], *Doo-Man Chun* [a, *], *and A. G. Abd-Elrahim* [a, b]

[a] *School of Mechanical Engineering, University of Ulsan, Ulsan, 44610, Korea*

[b] *Physics Department, Faculty of Science, Assiut University, Assiut 71516, Egypt*

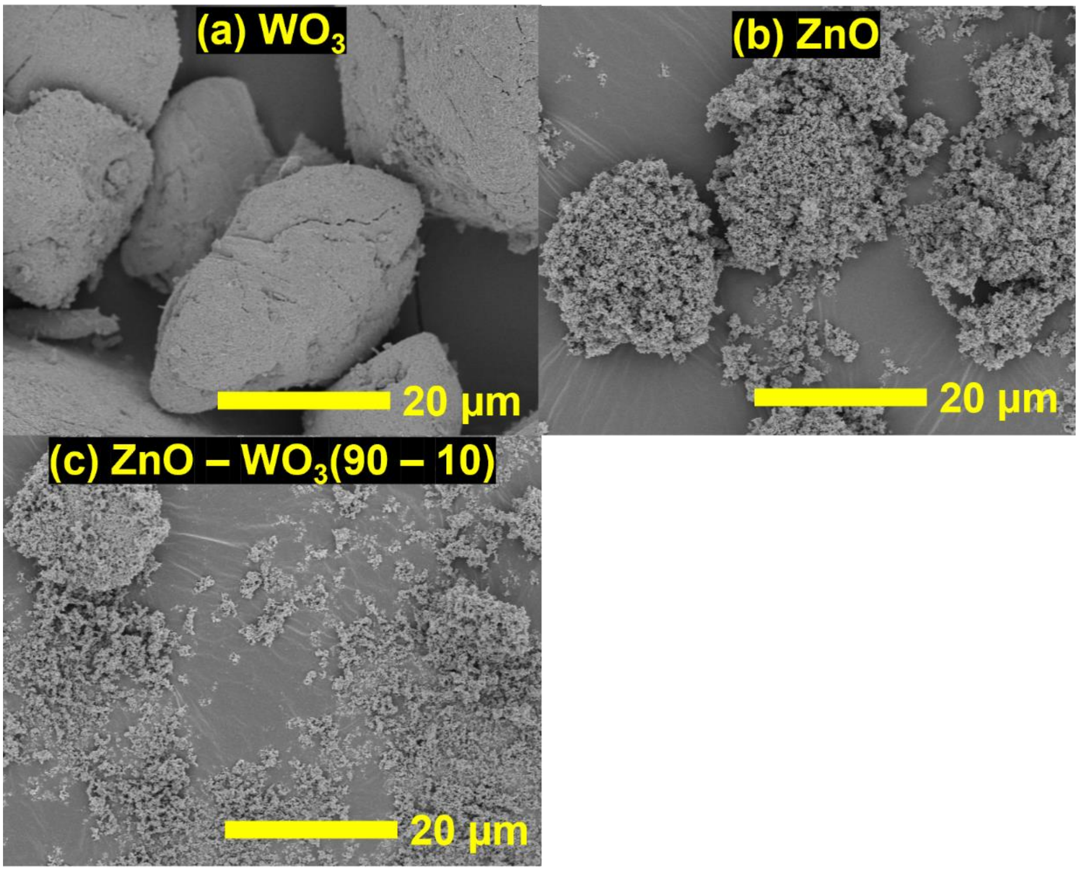

Figure S1: Scanning electron microscopy images of ZnO powder **(a)**, WO_3 powder **(b)**, and ZnO–WO_3 composite powder with 10% WO_3 content (c)

* Corresponding Author: dmchun@ulsan.ac.kr (Doo-Man Chun)

Table S1. The average consumable time for different material coatings in different techniques.

**Coating Techniques**	**Material**	**Average Consumable Time (Hours)**	**Ref**
Nanoparticle Deposition System (NPDS)	ZnO-WO_3	0.5	Our work
Sol-gel method+ Hummer's method	ZnO – Graphene Oxide	6.5	[1]
Aqueous chemical method	ZnO	11.3	[2]
Hydrothermal and cation exchange method	ZnO/$CuInS_2$	16	[3]
Solvothermal method	ZnO-WO_{3-x}	21	[4]

References:

[1] M. Ghorbani, H. Abdizadeh, M. Taheri, M.R. Golobostanfard, Enhanced photoelectrochemical water splitting in hierarchical porous ZnO/Reduced graphene oxide nanocomposite synthesized by sol-gel method, Int. J. Hydrogen Energy. 43 (2018) 7754–7763. https://doi.org/10.1016/j.ijhydene.2018.03.052.

[2] R. Lv, T. Wang, F. Su, P. Zhang, C. Li, J. Gong, Facile synthesis of ZnO nanopencil arrays for photoelectrochemical water splitting, Nano Energy. 7 (2014) 143–150. https://doi.org/10.1016/j.nanoen.2014.04.020.

[3] Y. Li, Z. Liu, Y. Wang, Z. Liu, J. Han, J. Ya, ZnO/CuInS2 core/shell heterojunction nanoarray for photoelectrochemical water splitting, Int. J. Hydrogen Energy. 37 (2012) 15029–15037. https://doi.org/10.1016/j.ijhydene.2012.07.117.

[4] Y. Chen, L. Wang, R. Gao, Y.C. Zhang, L. Pan, C. Huang, K. Liu, X.Y. Chang, X. Zhang, J.J. Zou, Polarization-Enhanced direct Z-scheme ZnO-WO3-x nanorod arrays for efficient piezoelectric-photoelectrochemical Water splitting, Appl. Catal. B Environ. 259 (2019) 118079. https://doi.org/10.1016/j.apcatb.2019.118079.
